# Efficiency of CD19 chimeric antigen receptor-modified T cells for treatment of B cell malignancies in phase I clinical trials: a meta-analysis

**DOI:** 10.18632/oncotarget.5582

**Published:** 2015-09-10

**Authors:** Tengfei Zhang, Ling Cao, Jing Xie, Ni Shi, Zhen Zhang, Zhenzhen Luo, Dongli Yue, Zimeng Zhang, Liping Wang, Weidong Han, Zhongwei Xu, Hu Chen, Yi Zhang

**Affiliations:** ^1^ Biotherapy Center, the First Affiliated Hospital of Zhengzhou University, Zhengzhou, Henan, China; ^2^ Department of Hematology and Oncology, Beth Israel Deaconess Medical Center, Harvard Medical School, Boston, Massachusetts, United States; ^3^ Department of Oncology, the First Affiliated Hospital of Zhengzhou University, Zhengzhou, Henan, China; ^4^ Center for Eye Research Australia, Royal Victorian Eye and Ear Hospital, University of Melbourne, Melbourne, Australia; ^5^ Comprehensive Cancer Center, the Ohio State University, Columbus, Ohio, United States; ^6^ Department of Immunology, Harvard Medical School, Boston, Massachusetts United States; ^7^ Molecular & Immunological/Bio-Therapeutic Department, Institute of Basic Medicine, Chinese PLA General Hospital, Beijing, China; ^8^ Department of Gastroenterology, Pennsylvania Hospital, University of Pennsylvania, Philadelphia, Pennsylvania, United States; ^9^ Department of Hematopoietic Stem Cell Transplantation, Affiliated Hospital to Academy of Military Medical Science, Beijing, China; ^10^ Engineering Key Laboratory for Cell Therapy of Henan Province, Zhengzhou, Henan, China; ^11^ School of Life Sciences, Zhengzhou University, Zhengzhou, Henan, China

**Keywords:** CD19, chimeric antigen receptor, B cell malignancies, meta analysis, efficiency

## Abstract

Chimeric antigen receptor (CAR) modified T cells targeted CD19 showed promising clinical outcomes in treatment of B cell malignances such as chronic lymphocytic leukemia (CLL), acute lymphoblastic leukemia (ALL) and other indolent lymphomas. However, the clinical benefit varies tremendously among different trials. This meta-analysis investigated the efficacy (response rates and survival time) of CD19-CAR T cells in refractory B cell malignances in Phase I clinical trials. We searched publications between 1991 and 2014 from PubMed and Web of Science. Pooled response rates were calculated using random-effects models. Heterogeneity was investigated by subgroup analysis and meta-regression. Fourteen clinical trials including 119 patients were eligible for response rate evaluation, 62 patients in 12 clinical trials were eligible for progression-free survival analysis. The overall pooled response rate of CD19-CAR T cells was 73% (95% confidence interval [CI]: 46-94%). Significant heterogeneity across estimates of response rates was observed (*p* < 0.001, I2=88.3%). ALL patients have higher response rate (93%, 95% CI: 65-100%) than CLL (62%, 95% CI: 27-93%) and lymphoma patients (36%, 95% CI: 1-83%). Meta-regression analysis identified lymphodepletion and no IL-2 administrated T cells as two key factors associated with better clinical response. Lymphodepletion and higher infused CAR T cell number were associated with better prognosis. In conclusion, this meta-analysis showed a high clinical response rate of CD19-CAR T cell-based immunotherapy in treatment of refractory B cell malignancies. Lymphodepletion and increasing number of infused CD19-CAR T cells have positive correlations with the clinical efficiency, on the contrary, IL-2 administration to T cells is not recommended.

## INTRODUCTION

Immunotherapy of cancer has shown a longer time to remission and complete cures in animal studies and clinical trials [1]. Adoptive immunotherapy using chimeric antigen receptor (CAR) modified T cells is a promising strategy developed in recent decades. CARs are artificial engineered receptors that can target special tumor cell surface antigen, activate T cells and further enhance T cell function MHC-independently. Objective tumor responses were reported in patients with acute lymphoblastic leukemia (ALL), chronic lymphocytic leukemia (CLL), and other indolent lymphomas after infusing autologous or allogeneic T cells genetically modified with CD19-CARs [2–16].

CD19 is an antigen expressed restrictively to normal and malignant B cells but not to other normal myeloid, erythroid, megakaryocytic, or multilineage bone marrow progenitor cells [17]. Therefore, CD19 is an attractive target for immunotherapies against B-cell malignancies. Compared with regular chemotherapies, application of CD19-CAR T cells showed better clinical outcomes and prognosis in treating refractory B cell malignancies. A complete remission rate of 90% and sustained remissions of up to 2 years has been reported in a clinical trial about CD19-CAR T cell therapies in patients with relapsed or refractory ALL [14]. In a recent published clinical trial of CD19-CAR-T cells in treatment of CLL patients, 3 of 4 patients achieved complete remission lasted for 23 months [7]. They also reported 8 of 11 lymphoma patients achieved remission lasted for 23 months. However, the clinical benefit varies broadly among different trials. In Savoldo's study, only two lymphoma patients achieved stable disease for 3 and 10 month but followed with disease progression [13]. In Cruz's study, only half patients achieved completed response and partial response [3]. These variations among studies might due to the different execute procedures such as the design of CAR structure, methods to introduce CAR into T cells, original T cell sources (autologous or allogeneic), T-cell culture conditions, lymphodepletion regimen, cytokine supports for T cell infusion, CAR T cell infusion schedule and dosage of CAR T cell. However, the key factor for better efficiency still remains unclear. A systematic review has examined the efficacy of CD19-CAR T cell therapies but the result was limited because only 5 clinical trails included in this review [18]. In this meta-analysis, we aimed to investigate the efficiency of CD19-CAR T cells immunotherapy on current published Phase I clinical trials. We also explored the factors affected the efficiency of CAR T cells immunotherapy using meta-regression analysis.

## RESULTS

### Basic information

**Figure 1:**
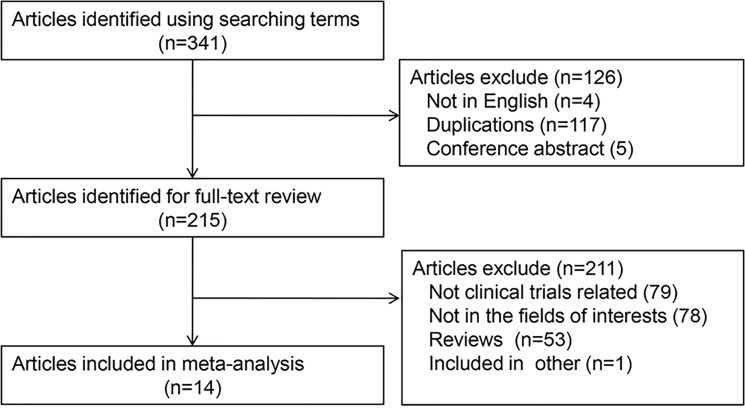
Flow diagram of study selection process

After duplicated literatures and meeting abstracts removed, we found 215 literatures related with CD19-CAR T cell research. Two authors identified articles eligible for further review by screen tittles and abstracts. We also found the clinical trial study literatures from the reviews. Finally, we identified 14 CD19-CAR T cell-based clinical trials for further review and analysis (Figure 1).

### Clinical trial and patient clinical characteristics

Our study included 14 Phase I clinical trials and 131 relapse or refractory B cell malignancies patients (73 ALL patients, 27 CLL patients, and 31 lymphoma patients) received CD19-CAR T cell immunotherapy [2–16]. The 31 lymphoma patients included 12 diffuse large B-cell lymphoma patients, 7 follicular lymphoma patients, 4 mantle cell lymphoma patients, 4 primary mediastinal B-cell lymphoma patients, 1 small lymphocytic lymphoma patients, 2 splenic marginal zone lymphoma patients and one patient without detailed subtypes. One patient died soon after infusion and another patient died because of influenza. Eight patients had no objective disease responses. Two patients lost to follow up. Therefore, 119 patients were eligible for the response rate evaluation. Since two of the clinical trials didn't present individual prognosis information, only 62 patients from 12 clinical trials were eligible for progression-free survival analysis.

### Clinical treatment strategy

The CAR design and manufacturing process are summarized in Table 1. Among these 131 patients, 2 patients in one clinical trial were treated with First-generation CAR T cells. Six patients in another clinical trial were given with both First-generation CAR T cells and Second-generation CAR T cells. Other patients were all administered with Second-generation CAR T cells. Eighteen patients in two clinical trials infused with allogeneic T cells derived from healthy donors, and all other patients infused with autologous T cells. Electroporation, lentivirus, gammaretrovirus and retroviruses are used to introduce CAR constructs into T cells. Most of the patients received lymphodepletion before CAR T cell infusion except 32 patients. There were 3 clinical trials didn't administrate lymphodepletion to all the patients. IL-2 was admitted to T cell culture or patients as lymphodepletion regimen. There are 72 patients without any IL-2 treatment in T cell culture or lymphodepletion regimens. The medians (range) of total infused T cell number and CAR^+^ T cell number were 2.4 ×10^8^ (8.76×10^6^-1.92×10^10^) and 1.5×10^8^ (1.4×10^7^-1.1×10^10^). (Data in cells/kg or cell/m^2^ were multiplied by 60kg or 1.73m^2^ respectively.)

**Table 1:** Clinical trial characteristics

No.^a^	Vector	T cell Original	Cell culture	Transfection method	T Cell treatment	CAR T cell persistence	Diagnosis	Lymphodepletion	Dose^b^	Response	Ref
2	IgG-CD4+ CD3	autologous	3 month	Electroporation	OKT3 + IL-2 +irradiated LCL feeders	1 day	lymphoma	Fludarabine IL-2	10^8^−10^9^/m^2^	2PD	9
1	CD28+ CD28 and CD3	autologous	18-26 day	Lentivirus	OKT3 + IL-2 +irradiated LCL feeders	36 weeks	lymphoma	Cyclophosphamide Fudarabine IL-2	1-3 ×10^8^	PR	12
9	CD28 + CD28+ CD3	autologous	6-18 day	Gammaretrovirus	OKT3 + IL-2	5-6 weeks	8CLL 1ALL	4 NA, Cyclophosphamide	0.4-3.0 ×10^7^/kg	3NE^c^, 1Died, 1CR, 1PD, 3SD	10
3	CD8-CD8 + 4-1BB + CD3	autologous	10 day	Lentivirus	CD3/28 beads	6 month	CLL	Pentostatin cyclophosphamide	1.46 × 10^5^/kg 1.0-1.6 × 10^7^/kg	2CR, 1PR	6
6	IgG-CD28 + CD3/CD28-CD3	autologous	6-8 day	Gammaretrovirus	OKT3 + IL-2	6 weeks	lymphoma	NA	2-10 ×10^7^/m^2^	6PR	13
8	CD28 + CD28+ CD3	autologous	24 day	Gammaretrovirus	OKT3 + IL-2	< 20 day or 8 weeks	4lymphoma 4CLL	Cyclophosphamide, Fudarabine, IL-2	0.3-2.8×10^7^/kg	1Died, 5PR, 1CR, 1SD	11
5	CD28 + CD28+ CD3	autologous	14 day	Gammaretrovirus	CD3/28 beads	3-8 weeks	ALL	Cyclophosphamide	1.4-3.2 ×10^8^/kg	5CR	15
8	IgG-CD28 + CD28+ CD3	allogeneic	5-6 week	Gammaretrovirus	Ad. Pp65+ EBV-LCLs+ IL-2	1-12 weeks	ALL	NA	1.5-12× 10^7^/m2	1CR, 1PR, 2CCR, 1SD, 3PD	3
2	CD8-CD8+ 4-1BB+ CD3	autologous	10 day	Lentivirus	CD3/28 beads	6 month	ALL	NA or Cyclophosphamide	0.14-1.2 × 10^7^/kg	2CR	4
10	CD28 + CD28+ CD3	allogeneic	8 day	Gammaretrovirus	OKT3 + IL-2	1 month	4 CLL 6 lymphoma	NA	1 × 10^6^ /kg	6SD, 2PD, 1CR, 1PR	2
11	lgG+ CD 28	autologous	14 day	Gammaretrovirus	CD3/28 beads	2-3 month	ALL	Cyclophosphamide	3 × 10^6^/kg	9CR, 2NE	16
15	CD28+ TCR	autologous	10 day	Gammaretrovirus	OKT3 + IL-2	<75 day	4 CLL, 11 lymphoma	CyclophosphamideFudarabine	5 × 10^6^/kg	4PR, 8CR, 1SD, 2NE	7
21	lgG+ CD3+CD28	autologous	11 day	retroviruses	CD3/28 beads	68 day	20 ALL, 1 lymphoma	Fudarabine Cyclophosphamide	1-3 × 10^6^/kg	3SD, 4PD, 13CR, CiR	8
30	CD8-CD8 + 4-1BB + CD3	autologous	10 day	Lentivirus	CD3/28 beads	up to 11 month	ALL	Fudarabine Cyclophosphamide	0.76-20 × 10 ^6^/kg	27CR, 3NE	14

a No.= patients in clinical trial received CD19-CAR T cells;

b Dose of infused T cells;

c NE= no response and no evaluation

### Meta-analysis of response rate of CD19-CAR T cell in patients with refractory B cell malignancies

**Figure 2:**
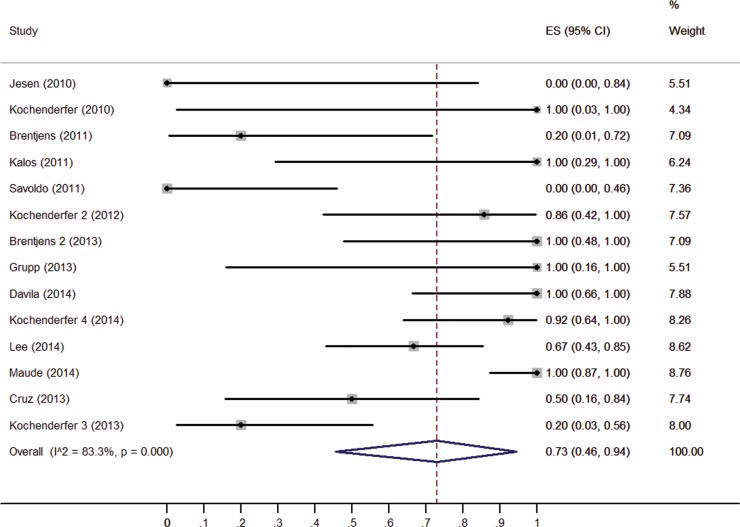
Forest plot for response rates and confidence intervals in each study and the overall

The response rate of CD19-CAR T cells in each clinical trial varied widely, from 0.0% [9] to 100.0% [4, 6, 12, 14–16]. Figure 2 shows the overall estimate of response rate and 95% confidence interval (CI) from the individual studies. Meta-analysis of all 14 studies yielded an overall pooled response rate of 73% (95% CI: 46–94%), with substantial heterogeneity observed (I^2^ = 83.3, *χ*^2^ = 77.63, *P* < 0.0001).

### Sources of heterogeneity

Both Begg's and Egger's regression asymmetry test showed no evidence of substantial publication bias (*P* = 0.260 for Begg's test; *P* = 0.102 for Egger's test). Then meta-regression analysis was performed based on CAR T cell protocols including T cell origin, T cell culture time, IL-2 administration to T cell culture, lymphodepletion before T cell infusion, IL-2 administration to patients, infused CAR T cell number and CAR T cell persistence time. Univariate meta-regression analysis showed that lymphodepletion, no IL-2 administration to T cells and T cell persistence more than 2 months positively associated with CD19-CAR T cells clinical responses (Table 2). Multivariable meta-regression analyses showed that lymphodepletion (*P* = 0.017) and no IL-2 administration to T cells (*P* = 0.017) were associated with heterogeneity.

**Table 2:** Univariate and Multivariable meta-regression analysis

Factors at study level	No. of Study	Response Rate (95% CI)	Univariate Meta-regression analysis		Multivariable Meta-regression analysis	
			Coefficients (95% CI)	*P* value	Coefficients (95% CI)	*P* value
**IL-2 administration to cell**						
Yes	9	0.43(0.10, 0.79)	0	***0.002***	0	***0.017***
No	6	0.98 (0.81, 1.00)	0.437 (0.164, 0.710)		0.302 (0.055, 0.550)	
**IL-2 administration to patient**						
Yes	3	0.64 (0.00, 1.00)		0.904	-	
No	12	0.74 (0.45, 0.96)	0.028 (−0.434, 0.491)		-	
**Lymphodepletion**						
Yes	10	0.88 (0.65, 1.00)	0	***0.002***	0	***0.017***
No	5	0.32 (0.01, 0.74)	−0.415 (−0.730, −0.172)		−0.347 (−0.632, −0.062)	
**T cell origin**						
Autologous	12	0.80 (0.52, 0.99)		0.054	-	
Allogeneic	3	0.33 (0.08, 0.64)	−0.397 (−0.800, −0.007)		-	
**T cell culture time**						
≥ 2weeks	7	0.74 (0.35, 0.99)	0	0.834	-	
< 2 weeks	8	0.72 (0.33, 0.99)	−0.037 (−0.384, 0.310)		-	
**Total CAR T cells**						
cells > 10^8^	10	0.84 (0.57, 1.00)	0	0.065	-	
cells < 10^8^	5	0.39 (0.00, 0.98)	−0.316 (−0.652, 0.020)		-	
**T cell persistence time**						
≥ 2 months	6	0.99 (0.80, 1.00)	0	***0.014***	0	0.912
< 2 months	9	0.48 (0.15, 0.82)	−0.378 (−0.680, −0.076)		0.014 (−0.226, 0.253)	

**Figure 3:**
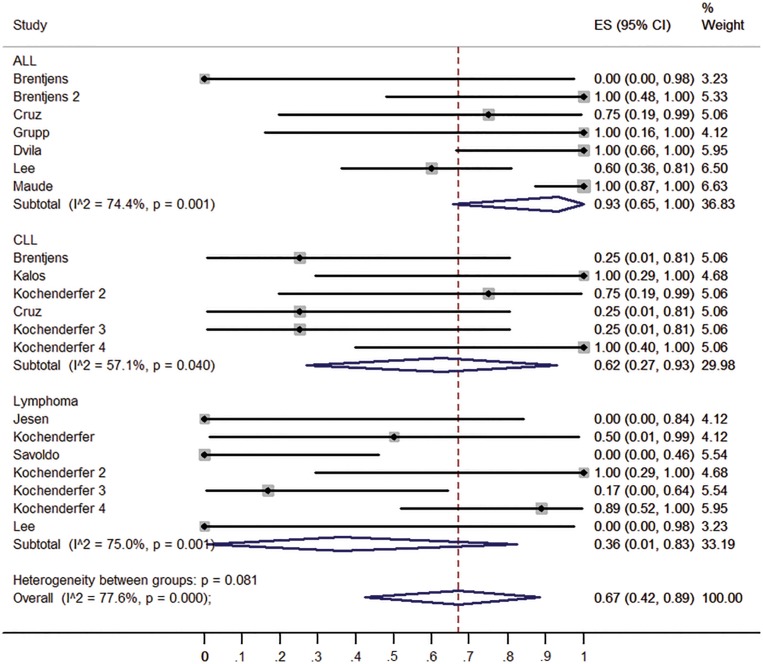
Forest plot for response rates and confidence internals in ALL, CLL and lymphoma patients

**Figure 4:**
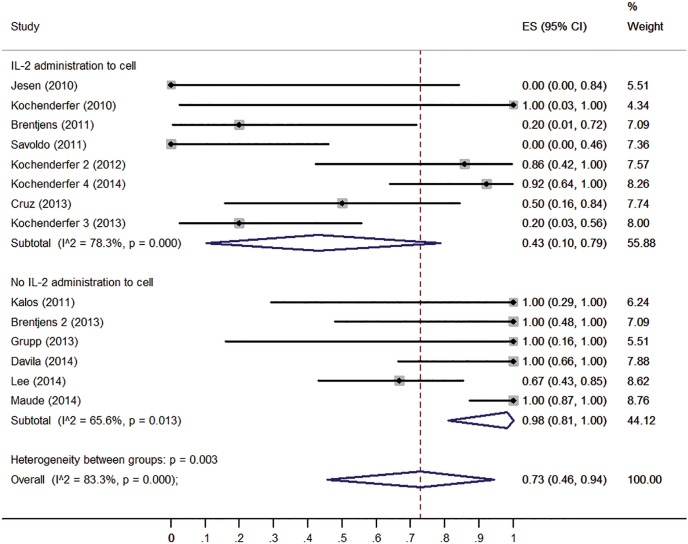
Forest plot for response rates and confidence internals in patients received IL-2 administrated T cells and patients received no IL-2 administrated T cells

**Figure 5:**
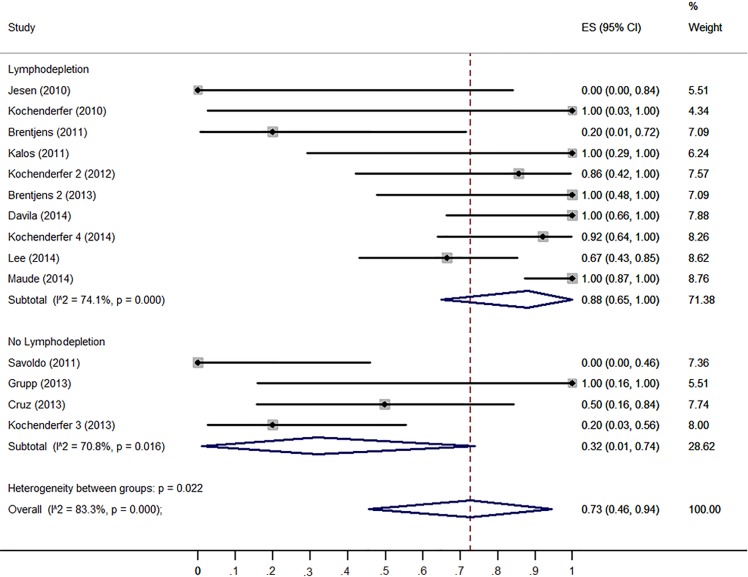
Forest plot for response rates and confidence internals in patients received lymphodepletion and patients without lymphodepletion

To confirm the results of the meta-regression, subgroups were analyzed. Firstly, we compared the clinical responses among different malignancies type (ALL, CLL and lymphoma). ALL patients have higher response rate (93%, 95% CI: 65-100%) than CLL patients (62%, 95% CI: 27-93%) and lymphoma patients (36%, 95% CI: 1-83%) (Figure 3). Patients received no IL-2 administrated T cells had higher response rate (98%, 95% CI: 81-100%) than those received IL-2 administrated T cell (43%, 95% CI: 10-79%) (Figure 4). Patients received lymphodepletion regimen had higher response rate (88%, 95% CI: 60-100%) than patients without lymphodepletion regimen (32%, 95% CI: 1-74%) (Figure 5). Results of other non-significant difference subgroups analysis were shown in supplemental figures (Figure s1–s5) and all the detailed data were list in Supplemental Table 1.

### Patient prognosis

**Figure 6:**
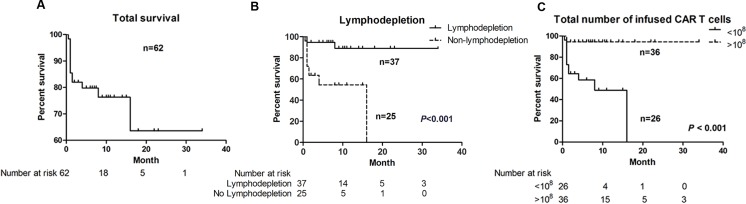
Progression-free survival (PFS) curves **A.** the PFS for total 62 patients; **B.** patients benefited from lymphodepletion; **C**. patients benefited from more than 10^8^ infused total CAR T cells.

The 6-month and 1-year PFS for total 62 patients were 80.0% and 76.3% respectively (Figure 6A). The median interval of PFS was 7.0 months. Only lymphodepletion and infused CAR^+^ T cell number were associated with better prognosis (Figure 6B, 6C). The 6-month PFS for patients administrated with lymphodepletion regimen before T cell infusion was 94.6%, significantly higher than 54.5% in patients without lymphodepletion (*P* < 0.001). The 6-month PFS for patients infused more than 10^8^ CAR^+^ T cell was 94.4%, significantly higher than 58.6% in patients infused less than 10^8^ CAR^+^ T cell ( *P* < 0.001). The survival curves of other factors were list in Supplemental Figure 6. Cox proportional hazards regression model showed that lymphodepletion was independently associated with better prognosis (Table 3).

**Table 3:** Lymphodepletion as an independent factor better prognosis by Cox Regression Model

Factors at study level	B	SE	Wald	df	Sig.	Exp(B)	95% CI for Exp (B)	
							Lower	Upper
IL-2 administration to Patient	−2.104	1.522	1.911	1	0.167	0.122	0.006	2.409
**Lymphodepletion**	3.429	1.519	5.097	1	0.024	30.846	1.572	605.339
T cell origin	−0.986	1.016	0.943	1	0.332	0.373	0.051	2.730
T cell culture time	−0.673	1.299	0.268	1	0.604	0.510	0.040	6.507
Total CAR T cells	1.333	0.911	2.140	1	0.143	3.793	0.636	22.629
T cell persistence time	0.743	1.153	0.415	1	0.519	2.101	0.219	20.127
Disease Type	−0.104	0.744	0.020	1	0.889	0.901	0.210	3.870

Note: IL-2 administration to cell was excluded from Cox regression because of the convergence.

## DISCUSSION

The response rates of CD19-CAR T cell in refractory B cell malignances varied widely. In current meta-analysis, the overall pooled response rate of CD19-CAR T cells in refractory B cell malignancies was 73% (95% CI: 46-94%). We also showed that lymphodepletion, no IL-2 administration to T cells and T cell persistence more than 2 months positively associated with CD19-CAR T cells immunotherapy clinical response.

CD19-CAR T cells have shown effective outcomes in B cell malignancies. Compared with the regular chemotherapies which is lower than 40% response rate [19–21], CD19-CAR T cell immunotherapy was competitive for treatment of patients with refractory B cell malignancies. The response rate of CD19-CAR T cell varied in different B cell malignancies, with higher response rate in ALL than CLL or lymphomas. The lower response rate in CLL and lymphomas might due to the host T-cell defects, and the strong inhibitory effects from tumor microenvironment [1, 22–25]. In two recent published clinical trials most of the ALL patients were adolescents aged less than 25 years old [8, 14]. Compared with regular regimen for adult ALL patients, adolescents and young adults could achieve better outcome from pediatric regimen [26]. However, whether ALL patients with younger age benefit more from CD19-CAR T cell needs further investigation.

CAR T cell immunotherapy is a multiple-step clinical practice with strict quality control clinical process. Lymphodepletion was administrated before the T cell infusion in most of the trials. Our meta-analysis showed that patients received lymphodepletion regimen had higher response rate than patients without lymphodepletion regimen. Multivariable meta-regression analyses also showed lymphodepletion was associated with higher response rates. Moreover, the survival analysis showed that patients received lymphodepletion before T cell infused had better prognosis than patients without lymphodepletion (*P* < 0.001). All these results suggested that lymphodepletion was a critical factor for better clinical outcomes. Lymphodepletion before T cell infusion aims to remove suppressor regulatory T cells, eliminate some cytokines dependently lymphoid cells to extend the infused CAR T-cell persistence and expansion *in vivo* [18, 27–28]. The meta-regression also verified CAR^+^ T cell persistence time *in vivo* associated with better clinical response but not independently. Considering the response rate and microenvironments differences between ALL and CLL/lymphomas, we suggest that lymphodepletion was important for the clinical outcomes of CAR T cells immunotherapy through regulation on tumor microenvironment. However, we didn't find any difference between the five lymphodepletion regimens by meta-regression. This may due to the small number of trials involved in the meta-analysis and too many variations in the regression setting. With more precious clinical trials about CD19-CAR T for B cell malignancies, the detailed association between lymphodepletion and clinical outcomes will be elaborated in the future.

It is recommended that cytokines can be useful in improving the expansion of First generation CAR, and potentially benefit on Second/Third generation CAR T cells. As cytokine support, IL-2 can promote T cell expansion *in vitro* to improve treatment outcome in CD19-CAR T cell immunotherapy [29]. But our findings showed that no IL-2 administration to T cells associated with better clinical response. Compared with First-generation CARs with only a CD3 intracellular signaling domain, Second-generation CARs include another single costimulatory domain derived from either CD28 or 4-1BB [1, 30]. Second-generation of CARs showed superior outcomes in both animal study and clinical trials [1, 30]. First generation of CARs failed to elicit robust cytokine response, including IL-2 can support T cell expansion upon repeated exposure to antigen [30]. The key advantage of Second generation CARs was the induction of IL-2 secretion and T cell proliferation upon CAR cross-linking [30]. Among the fourteen trials in this Meta-analysis, thirteen clinical tirals used the Second generation CAR T cells, and part of them didn't use IL-2 during CAR T cell culture. In these clinical trials without IL-2 administration to T cells, anti-CD3/anti-CD28 mAb-coated magnetic beads were used to stimulate T cell expansion. CD28 stimulation may play a positive role for CAR T cell proliferation. For proliferation, CD3 antibody can provide an initial activation signal, but proliferation is dependent on co-stimulatory CD28 [31]. Both CD4^+^ and CD8^+^ T cells contribute to the *in vivo* expansion of CAR^+^ T cells [13]. CD4^+^ T cells respond well to CD3/CD28 stimulation. Moreover, IL-2 might limit clonally expansion and the accumulation of antigen-speciﬁc effector T cells by promoting activation-induced cell death [32–33], but CD3/CD28 can increase T cell proliferation without provoking early cell death [34]. The difference between patients received IL-2 administrated T cell and no IL-2 administration T cells may come from either IL-2 or anti-CD28. However, the difference between IL-2 stimulation and CD3/CD28 stimulation still needs more verification. There was no difference between patients administrated with IL-2 or not in lymphodepletion regimen.

On-target/off-tumor effect and cytokine-released syndrome (CRS) are two major safety concerns for CAR T cell immunotherapy. On-target but off-tumor effects result from the immune response in normal cells with the CAR-targeted antigens. B-cell aplasia is an on-target but off-tumor effect of CD19-CAR-directed therapies [10–11]. CRS can be caused by cytokine secretion in response to the activation of CAR T cells. CRS is often accompanied by macrophage activation syndrome, which is characteristic of hyperinflammation with prolonged fever, hepatosplenomegaly, and cytopenias [4]. Among these trials involved in this meta-analysis, the infusions were well tolerated without any immediate adverse side effects in two trials [3, 13]. But grade 3 and grade 4 severe adverse effects associated with CD19-CAR T cell infusion were reported in all the other twelve trials, much of the toxicity that occurred in these patients was because the elevations in inﬂammatory cytokines. The adverse effects, fever, rigors, and dyspnea were commom within the first 24 hours, but can be controlled either by reducing the dose or between IL-6 blocker tocilizumab treatment. On-target/off-tumor effect and B cell aplasia, was reported in six trials. However, lymphodepletion, total number of CAR T cells and T cell persistence might also correlate with the toxicities, but in this study we didn't evaluated the potential factors associated with toxicities.

To improve efficiency and reduce the toxicities, several new strategies are recruited for CAR T vectors. Inducible Caspase 9 (iCasp9) was integrated to CAR construction as “safety switch” to control the on-target/off-tumor toxicities [35]. The combination of CAR and a second chimeric costimulatory receptor can increase the tumor target specific and avoid side effects [36]. Modification CAR T cells to secrete IL-12 or using native virus-specific T cells to transducer with CAR vectors can exhibit longer persistence time of CD19-CAR T cells in microenvironment in order to enhance the efficiency [37–38]. CTLA-4 and PD-1 are two important immune checkpoints negatively regulating T cell activation. Blockade PD-1, PD-L1 or CTLA-4 can prolong the efficiency of activated T cell during immune reaction [39–40]. Combination of CD19-CAR T cells with PD-1/PD-1 or CLTA-4 antibodies has the possibility for better clinical outcomes.

In conclusion, our meta-analysis showed a high clinical response rate of CD19-CAR T cells in refractory B cell malignancies than regular chemotherapies. The meta-analysis also found lymphodepletion regimen as a key factor associated with better clinical responses. Lymphodepletion is recommended to clinical procedures in treatment of B cell malignancies using CD19-CAR T cell-based immunotherapy. We believe that combined the new technology development with the lessons from retrospective studies would lead to better clinical efficacy by applying CD19-CAR T cells in treating B cell malignancies.

## MATERIALS AND METHODS

### Search strategy and study eligibility criteria

We searched for articles published from Jan 1, 1991, to December 31, 2014 with key words “chimeric antigen receptor” combined by “AND” with “CD19” in both MEDLINE and Web of Science. Two authors (TF Z and CL) identified articles eligible for further review by screening tittles and abstracts. When a study was considered relevant, the article was reviewed thoroughly. Only literatures published in English and reported clinical trials with the application of CD19-CAR T cells in treatment of refractory B cell malignancies including B-ALL, B-CLL and lymphoma were eligible for further review.

### Literature screening

Gender, age, malignancies type, CAR design, gene transduction method, original T cell sources, T cell culture time, lymphodepletion, IL-2 administration for T cell culture and patient lymphodepletion regimen, total infused CAR^+^ T cell number, CAR^+^ T cell persistence time, patients’ response to CAR T cells and follow-up time were all collected from each study. The primary endpoint was the response to CAR T cells immunotherapy. Response was based on the cytologic immunological test or computed tomography scans reported by each trial. Patients died not because of malignancies, lost follow-up, and with no objective disease response were excluded for analysis. Patients with response to CAR T cells immunotherapy were divided to two group: positive response group (patients achieved complete response (CR) or partial response (PR)), and negative response group (patients achieved stable disease (SD), progress disease (PD)). The response rate was calculated by the percentage of patients achieved complete response and partial response. For detailed analysis, IL-2 administration and lymphodepletion were analyzed by “Yes” and “No”; T cell origin was analyzed by “Autologous” and “Allogeneic”; T cell culture time was analyzed by “≥ 2 weeks” and “< 2 weeks”; Total T cell culture time was analyzed by “cells > 10^8^” and “cells <10^8^”; T cell persistence time was analyzed by “≥ 2 months” and “< 2 months”. We assessed studies for quality on the basis of the Cochrane Collaboration's method for non-randomized studies [41].

### Statistic analysis

Metaprop is a statistical program implemented to perform meta-analyses of proportions in Stata13.0 (Stata Corp, College Station, TX) [42]. Metaprop implements procedures which are specific to binomial data allows computation of exact binomial and score test-based confidence interval. It provides appropriate methods for dealing with proportions close to 0 or 100%. By using Metaprop, no studies with 0% or 100% proportions were excluded from the meta-analysis. The Freeman-Tukey double arcsine transformation was used to compute the weighted pooled response rate.

We used the Cochran's Q test to assess between-study differences and the I^2^ statistic to quantify the proportion of observed inconsistency across study results not explained by chance. If the heterogeneity among trials were very large (I^2^ statistic>75%), the observed difference between the response rates cannot be entirely attributed to sampling error and other factors such as differences in study population, etc. could also contribute. Thus, a random effects meta-analysis was used to pool the response rates of CD19-CAR T cells in refractory B cell malignances in Phase I clinical trials. The pooled response rate describes the mean of the distribution of the estimated response rate.

Univariate meta-regression analyses were conducted to identify clinical factors associated with response rate. Next, we performed a multivariate meta-regression analysis on the individually significant factors from the univariate analysis. Potential interaction was also tested between potential predictors.

To study possible publication bias, we evaluated Contour-enhanced funnel plots. A deficiency in the base of the funnel with asymmetry indicates the presence of possible publication bias from unpublished small studies. On a contour-enhanced funnel plot, contours of statistical significance are overlaid on the funnel plot. Publication bias was also assessed by two formal tests: Begg's adjusted-rank correlation test and Egger's regression asymmetry test.

All the factors analyzed in univariate meta-regression analyses were evaluated for CD19 CAR T cell immunotherapy prognosis according to progression-free survival (PFS). The interval for PFS was defined as the time from CAR T cell infusion to disease progress. PFS curve was estimated by Kaplan-Meier method and compared by log-rank test between each factors analyzed in univariate meta-regression analyses. The Cox proportional hazards regression model was used to identify independent prognostic factors. A two-sided P value was considered as statistically significant.

## SUPPLEMENTARY MATERIAL FIGURES AND TABLES




