# Supplementary material for: Efficiency of CD19 chimeric antigen receptor-modified T cells for treatment of B cell malignancies in phase I clinical trials: a meta-analysis
Source: Oncotarget. 2015 Sep 10;6(32):33961–71. doi: 10.18632/oncotarget.5582 (PMC4741817; doi:10.18632/oncotarget.5582)
Supplement: Supplementary file 1 [file oncotarget-06-33961-s001.pdf]

Efficiency of CD19 Chimeric Antigen Receptor-Modified T Cells for treatment of B Cell Malignancies in Phase I Clinical Trials: a Meta-Analysis

Supplementary Material

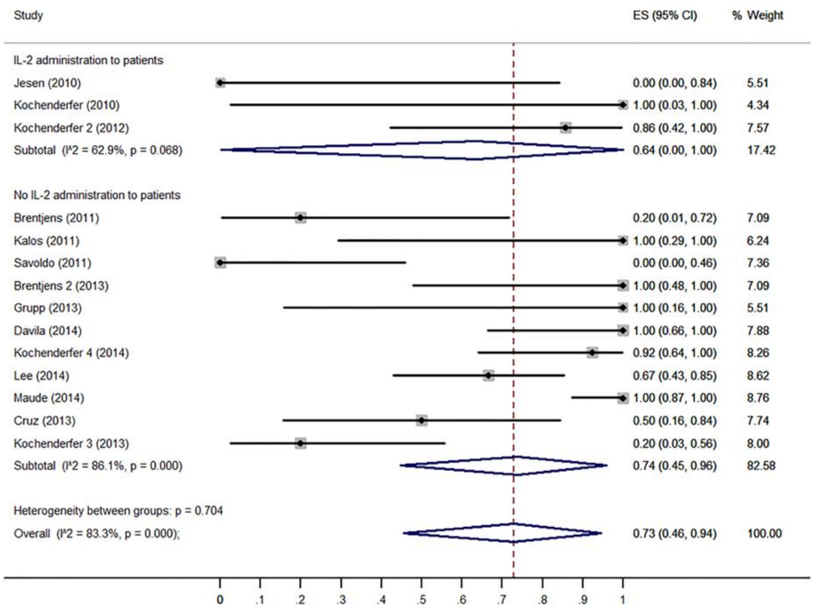

Supplemental Figure 1: Forest plot for response rate and confidence intervals in patients with/without IL-2 administration to patient in the meta-analysis.

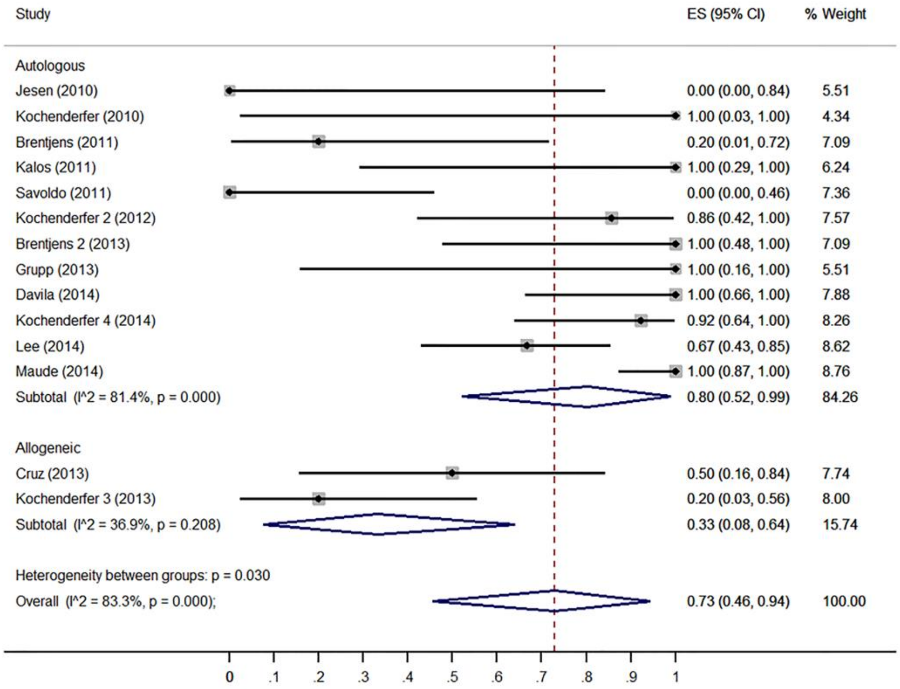

Supplemental Figure 2: Forest plot for response rate and confidence intervals in patients received different original T cell in the meta-analysis

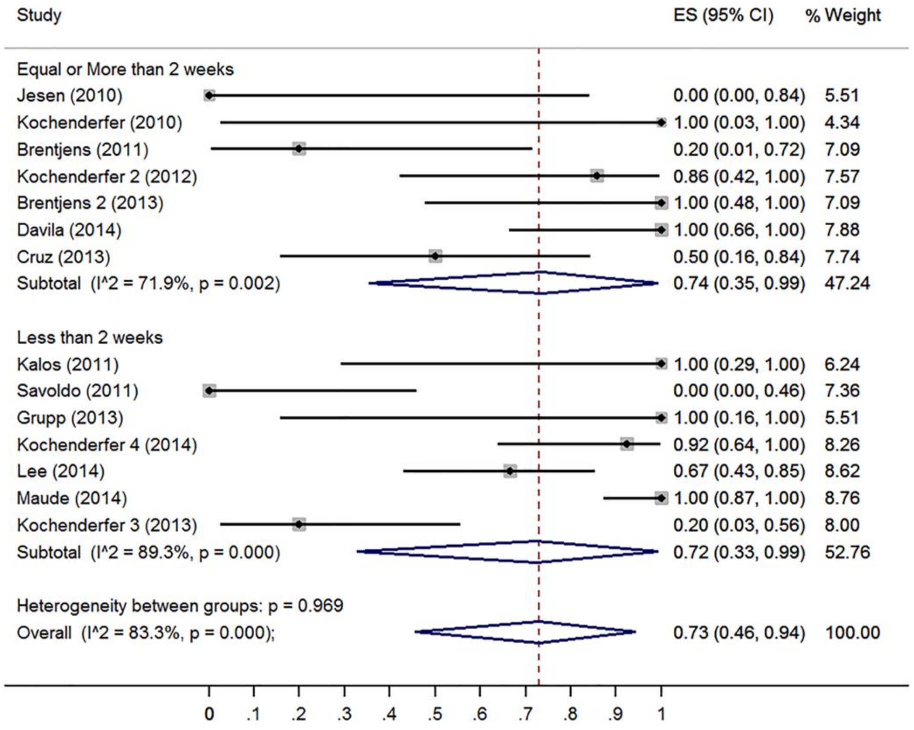

Supplemental Figure 3: Forest plot for response rate and confidence intervals in patients with different T cell culture time in the meta-analysis.

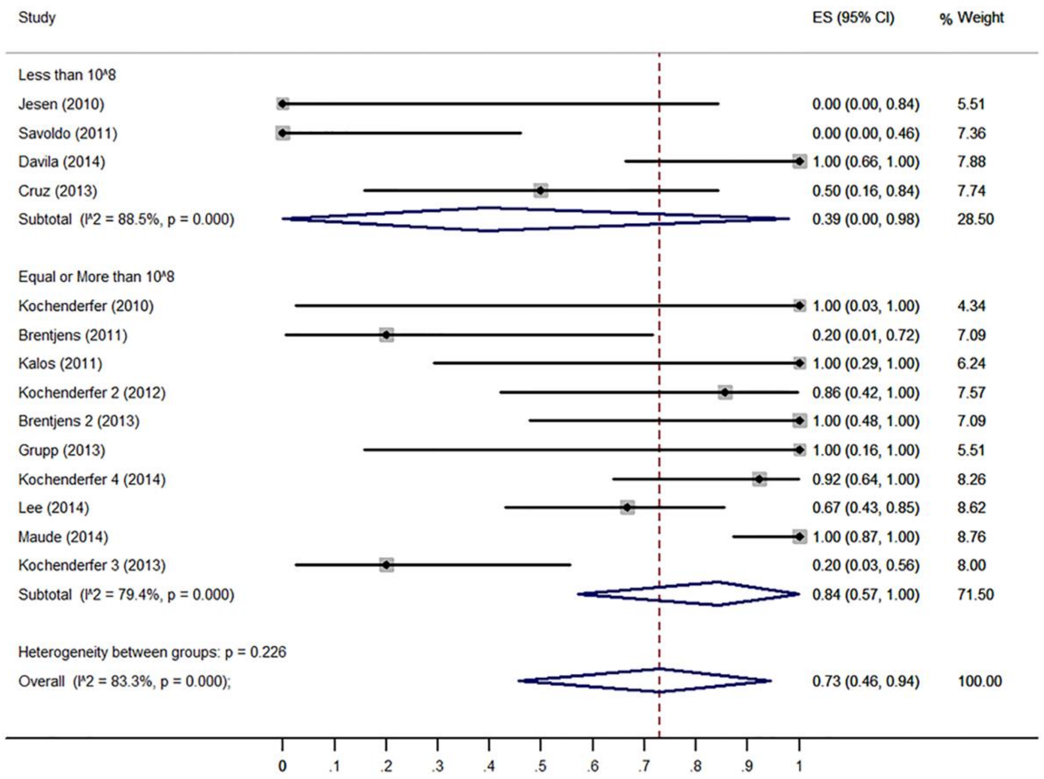

Supplemental Figure 4: Forest plot for response rate and confidence intervals in patients with different infused CAR T+ cell number in the meta-analysis.

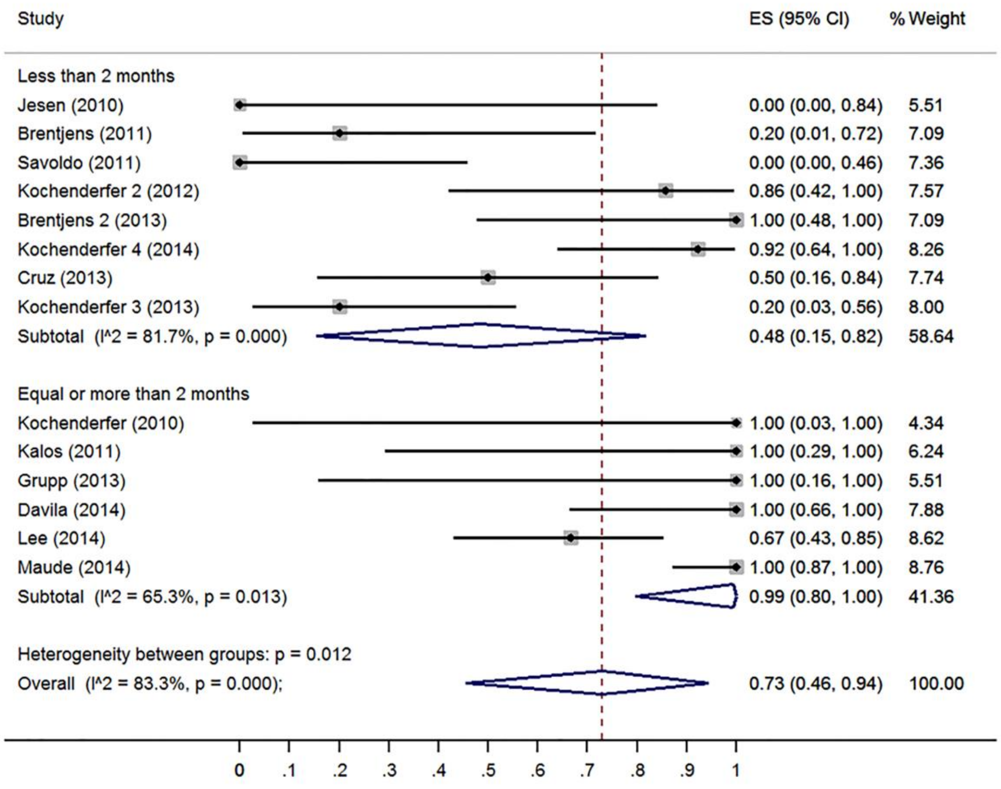

Supplemental Figure 5: Forest plot for response rate and confidence intervals in patients with different CAR T+ cell persistence time in the meta-analysis.

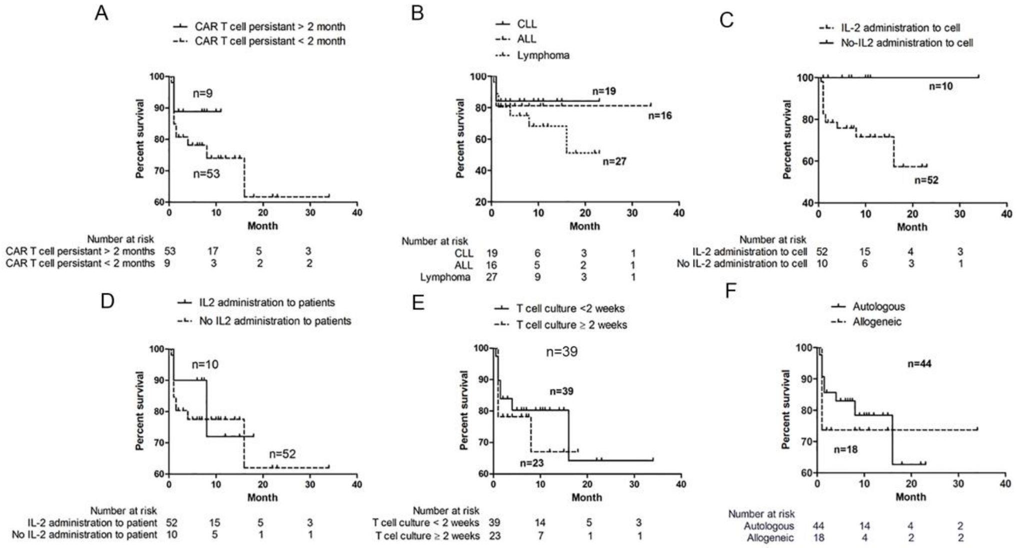

Supplemental Figure 6: Progression-free survival (PFS) curves.

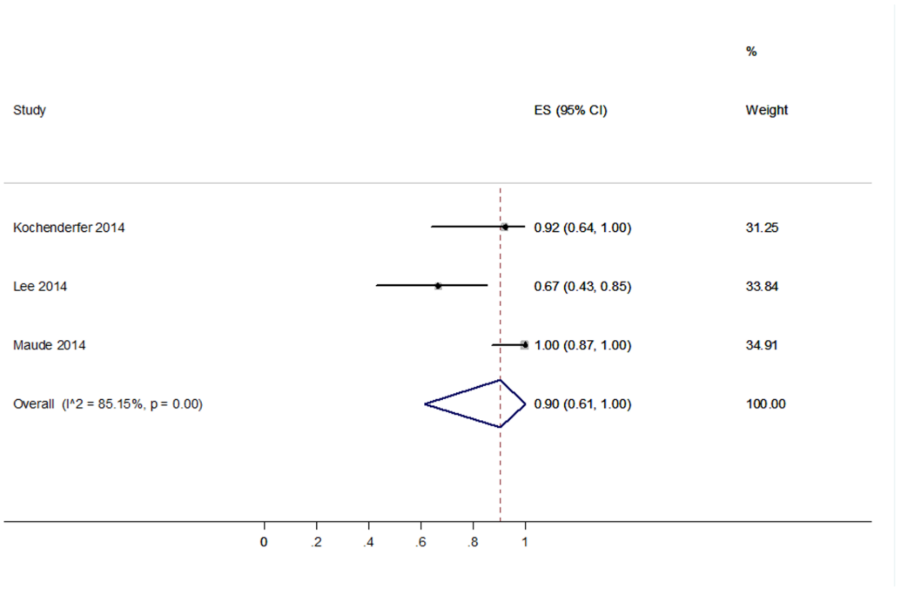

Supplemental Figure 7: Forest plot for response rates and confidence intervals in 3 studies with more than ten patients.

Supplemental Table 2: Meta-regression of the association between lymphodepletion regimens and clinical outcomes

Lymphodepletion regimen	Co-efficiency.	95% CI	P value
Fludarabine + IL-2	1.00	-0.31, 2.31	0.11
Cyclophosphamide + Fudarabine + IL-2	-0.12	-1.60, 1.42	0.86
Cyclophosphamide	-0.20	-1.61-1.21	0.740
Cyclophosphamide + Fudarabine	-0.13	-1.48, 1,21	0.82
Pentostatin + cyclophosphamide	NA*	NA*	NA*

Note: *There was only one trail in "Pentostatin + cyclophosphamide" group. The Meta regression can't show the pooled regression results for this group.
